# The relationship between perceived built environment and cycling or e-biking for transport among older adults–a cross-sectional study

**DOI:** 10.1371/journal.pone.0267314

**Published:** 2022-05-03

**Authors:** Tanja Brüchert, Paula Quentin, Gabriele Bolte

**Affiliations:** 1 Department of Social Epidemiology, Institute of Public Health und Nursing Research, University of Bremen, Bremen, Germany; 2 Health Sciences Bremen, University of Bremen, Bremen, Germany; 3 Faculty of Spatial Planning, Department of Urban and Regional Planning, TU Dortmund University, Dortmund, North Rhine-Westphalia, Germany; Southeast University, CHINA

## Abstract

The built environment plays a key role in promoting active mobility for healthy aging. Encouraging active mobility among older adults, however, can be especially challenging in more rural areas where distances tend to be longer and infrastructure is favoring car traffic. The association of older adults’ perception of attributes of the built environment with cycling and e-biking for transport was investigated. The potential moderating effects of age, sex, and mobility impairments were explored. A cross-sectional survey was conducted in towns and communities of <100,000 inhabitants, including 2,144 participants (mean age: 74; 53% men). Three different cycling outcomes (any cycling/e-biking, frequency (≥3 days/week) and amount (minutes/week)) were analyzed among all cyclists and e-bike users separately, resulting in six dependent variables. The impact of ten environmental attributes on these outcomes was analyzed in separate models. Overall, street connectivity, and the proximity and number of destinations were most important. Only street connectivity and traffic safety were related to minutes cycling or e-biking per week. Cycling amount was higher overall for e-biking but fewer environmental attributes showed an association compared to any cycling, regardless of bicycle type. Traffic safety was not associated with four out of the six cycling outcomes and was inversely associated with minutes cycling or e-biking. Density was not related to any of the variables investigated. Several interaction effects of sex, age, and mobility impairments were found. Further research is needed on environmental attributes influencing e-bike use, the most important types of destinations, and a more differentiated assessment of perceived traffic safety in older adults.

## Introduction

Worldwide, cycling for transport has come into focus of urban and transport planning as a solution to tackle climate challenges and congestion in cities [1]. The public health sciences have adopted the subject as a means to increase physical activity levels in the population. Physical activity has a wide range of positive health effects including improved cardiorespiratory fitness, reduced cancer risk, and increased wellbeing [2,3]. The WHO recommends 150 minutes of moderate-intensity physical activity per week to realize these benefits [4]. Cycling is classified as a moderately intensive activity [5]. A meta-analysis on cycling found a 10% risk reduction in all-cause mortality when WHO recommendations were met. However, the health benefits were greatest for the first one or two hours of cycling and then decreased [6]. This suggests that while reaching the WHO recommendations for physical activity is important, simply being active compared to not being active at all can have extensive health benefits.

Cycling has also great potential to reduce health inequalities as participation is supposed to be more equally distributed compared to sports and leisure activities, which are more popular in high-income and well-educated population groups [7]. When it is perceived as safe and comfortable, cycling is a cost-effective transport option that is more likely to be taken up by people who are not sufficiently active. In countries with high levels of cycling and safe cycling conditions, such as parts of Germany and the Netherlands, cycling is popular among both genders and across all age groups [8,9]. Cycling is also accessible to disabled people, and may be easier than walking for particular groups (e.g. with a hip problem). However, the design of the built environment needs to consider the needs of people with different levels of experience and ability to support safe cycling [10].

Given the rising number of older adults in many countries and the potential impacts on society and the health care sector the development of age-friendly environments, i.e. environments that support healthy ageing, is a major policy goal globally [11]. The neighborhood built environment, in particular, plays a key role in promoting active mobility for healthy aging [12]. However, this can be especially challenging in more rural areas where distances tend to be longer and car use is high among elderly people [13]. This reliance on car driving can become a problem with age as people may lose the ability to drive or may lack access to a vehicle. In fact, car use is declining with age [9].

Recent systematic reviews have found residential density, street connectivity, land use mix, pedestrian and cycling infrastructure, public transport, aesthetics, and crime and traffic safety to be associated with older adults’ overall physical activity as well as active mobility modes [14–16]. Most of this research, however, has been focusing on correlates of walking and the built environment. To the best of the authors’ knowledge, only three quantitative studies have examined the association between living environment factors and cycling in older adults [15,17]. Moreover, most of the studies–both quantitative and qualitative—on environmental features influencing physical activity were carried out in urban areas and almost exclusively outside of Europe [15,18]. These results are therefore not easily transferable due to different mobility cultures and diverse settlement patterns. Existing studies on infrastructure and active mobility in Germany lack a public health perspective [19]. Furthermore, the increasing popularity of the e-bike might be a promising development for public health that is yet largely unexplored. An e-bike is a bicycle which supports the rider with an electric motor up to a maximum of 250 watts when pedaling. Distances covered by e-bikes are on average four to eight kilometers longer than with conventional bikes [9]. The e-bike is especially popular among the older population [9,20,21]. E-bikes are also often used by people with health problems to maintain or increase personal fitness [22]. However, due to the speed of e-bikes, accidents can be serious. Although studies from China show that older e-bike users have less risky traffic behaviors than younger ones, such as running red lights [23], they face problems in case of balancing at very low speed or while static, mounting and dismounting, or with the ability to maneuver [10]. This emphasizes the importance of safe road conditions that take into account the needs of vulnerable groups.

Overall, little is known about the association of the built environment in less dense urban areas with cycling and e-biking for transport and how this association varies by age, sex, or mobility impairments [15]. As a consequence, the special needs of vulnerable groups, such as the elderly population with limited mobility functions, are not well considered in city and transport planning. Moreover, studies indicate that the perception of certain environmental attributes has a greater effect on cycling [24] and neighborhood satisfaction [25] than objectively measured factors. This is particularly true for perceived safety, which is considered a crucial determinant of cycling behavior [26,27]. As such, measures of interventions need to address peoples’ perception of their environment if they are to successfully promote cycling.

The aim of this study is to investigate the relationship between older adults’ perception of built environment attributes and cycling and e-biking for transport. Furthermore, an explorative approach is taken to analyze potential moderating effects of age, sex and mobility impairments with environmental attributes on cycling behavior.

## Material and methods

### Study design

Within the project *AFOOT—Securing urban mobility of an aging population* [28], a cross-sectional postal survey was conducted in the Metropolitan Region of Bremen-Oldenburg in the Northwest of Germany. The survey took place between May and September in 2019. The study was approved by the University of Bremen ethical committee (ethics vote 20181205).

### Study population

Study participants are people aged 65 and older. The age limit of 65 years was chosen because it is the usual retirement age and the population group aged 65 years and older is considered as older adults by the Federal Statistical Office in Germany. The study area includes eleven rural districts and two urban municipalities (each with fewer than 100,000 inhabitants) in the state of Lower Saxony. Sampling was carried out by the 117 residents’ registration offices of the included districts and municipalities. A total of 11,000 people were randomly selected. Total response rate was 20.56% (see S1 Fig for details).

### Measures

Validated and nationally or internationally established question modules were used in the questionnaire where suitable. The design of the questionnaire (length, font size, contrasts) was adjusted based on experience with the age group. The questionnaire was tested and discussed twice in focus groups with the target population (people aged 65 years and older) to adapt the questions to the German context and to reveal problems with length and complexity of the questionnaire. A specific reliability test of the questionnaire was not performed.

#### Socioeconomic variables

*Sex* was assessed as dichotomous variable (‘Male’, ‘Female’) and *age groups* were built with respect to quartiles (‘65–69 years’, ‘70–74 years’, ‘75–79 years’, ‘80 years and over’). Information about the relationship status were summarized as *marital status* with the categories ‘Married’, ‘Single’, and ‘Widowed’ and, additionally, *partner status* with the categories ‘Having a partner’ and ‘No partner’. Three categories emerged assessing *school education*: ‘High’ (A-levels, general or subject-specific qualification for a place at university or technical college qualification), ‘Middle’ (Secondary school/vocational secondary school–Polytechnic institute qualification, or class 10 (before 1965: class 8) or other school-leaving qualification (e.g. obtained abroad)) and ‘Low’ (Elementary school or Left school without qualification) [29]. Information about school education and professional education were summarized as *education* according to the International Standard Classification of Education (ISCED) [30] and classified into three categories: ‘Low’ (ISCED 0–2), ‘Middle’ (ISCED 3–4) and ‘High’ (ISCED 5–6). *Income* was assessed by asking participants to give information about their monthly net income and their household composition (number and age of household members). Following the guidelines of the OECD [31], the equivalized disposable income was calculated. With respect to the latest poverty line of Lower Saxony [32], three categories were used to classify the level of income: ‘Low’ (< 60% median), ‘Middle’ (≥ 60% median and ≤ median) and ‘High’ (> median). Living situation was collapsed into two categories ‘Own private home’ and ‘Other’ (which included, for example, living with others). *Country of birth* was analysed as a dichotomous variable (‘Germany’, ‘Other country’). Concerning the *area of residence*, participants were asked to name their municipality. Based on the classification of the Federal Institute for Research on Building, Urban Affairs and Spatial Development [33] the named municipalities were categorized into four groups: ‘Medium-sized town’ (20,000 - < 100,000 inhabitants), ‘Larger small town’ (10,000 - < 20,000 inhabitants), ‘Small town’ (5,000 - < 10,000 inhabitants or at least basic central function) and ‘Rural community’ (< 5,000 inhabitants).

#### Health variables

Participants reported their *self-rated health* on a 5-point scale [34]. Answers were dichotomized into ‘Very good/good’ or ‘moderate/bad/very bad’. To assess *mobility impairments*, multiple answers were possible: ‘Yes, I have a walking disability’–‘Yes, I have a visual impairment’–‘Yes, I have (an) other mobility impairment(s)’–‘No, I don’t have mobility impairments’. Answers were dichotomized into ‘None’ versus ‘At least one’. Use of a *walking aid* was dichotomized as ‘No’ or ‘Yes’ (if any of: Walking stick/ cane, Crutches, Walker, Walking frame, Quad walker, or Wheelchair).

#### Transport variables

*Car availability* assessed access to a car as a driver or passenger. Response categories were ‘Always’, ‘Sometimes’ or ‘Never’. For *bicycle ownership* participants were asked which of the following applied: ‘conventional bicycle’, ‘e-bike/pedelec’, ‘tricycle’, or ‘none of the three’. Multiple answers were possible.

#### Dependent variables

*Frequency of cycling for transport* was assessed by asking participants to indicate separately how often they usually use each of a conventional bicycle or an e-bike as a means of transport. Response options were: ‘Daily or almost daily’; ‘3–4 days a week’, ‘1–2 days a week’, ‘1–3 days per month’, ‘rarer’, or ‘never’. Respondents were classified into two categories: ‘≥3 days/week’ versus ‘<3 days/week’. To be able to compare any bicycle use with e-biking only, another outcome was defined to include only those people that either owned an e-bike or had no bicycle at all (classified as ‘never’ e-biking for transport). *Frequency of e-biking for transport* was created accordingly, with two levels: ‘≥3 days/week’ and ‘<3 days/week’.

To assess differences in the relationship between the perceived environment and cycling (regardless of the type of bicycle) as well as e-biking specifically, this procedure was applied to all outcomes (see S2 Fig for more details).

The *amount of cycling in minutes per week* was measured using the domain specific questions for transport activity from the International Physical Activity Questionnaire long form (IPAQ–LF). The IPAQ–LF has been found to have acceptable test-retest reliability [35]. The IPAQ-LF is designed for adults aged 15–69 years. As participants in this study are 65 years and older and even small amounts of activity may be beneficial for health in this group [6,36], the questions were modified to ask about trips of at least 5 minutes. The IPAQ–LF item used in the analyses distinguish between any cycling for transport and e-biking. The item assessed the number of days during the last week that were spent cycling for ≥5 minutes to get from place to place and the usual minutes spent doing so per day. *Minutes per week spent cycling* (whether conventional bicycle or e-bike, or both) or *e-biking* (e-bike use only) were calculated and treated as discrete variables. IPAQ data were truncated at the maximum value of 1260 min/week according to the IPAQ protocol [37].

In addition, dichotomous outcome measures were derived to represent *any cycling* that lasted for ≥5 minutes during a typical week (whether conventional bicycle or e-bike, or both) or *e-biking (e-bike users only)* for transport with the categories ‘Yes’ versus ‘No’.

In order to differentiate between bicycling for transport purposes and bicycling for leisure purposes, the questionnaire also addressed this issue, which is, however, not part of this research question.

#### Independent variables

Assessment of the environment was largely based on the Neighborhood Environment Walkability Scale (NEWS) questionnaire and scoring protocol [38] with modifications for the German context. The NEWS is the most frequently-used tool to measure perceived neighborhood environmental attributes [39].

*Residential density* was assessed asking about six types of residences in the participants’ neighborhood: detached single-family residences, townhouses or row houses of 1–3 stories, and apartment houses (1–3 stories, 4–6 stories, 7–12 stories, or more than 13 stories). Participants rated all types on a 5-point scale from ‘All’ to ‘None’. A score was calculated according to the NEWS scoring protocol.

*Land use mix* was assessed with the question: ‘About how long would it take to get from your home to the nearest facilities for the local supply and services listed below if you walked to them?’. There were four answer options (1–10 minutes, 11–20 minutes, 21–30 minutes, or 31+ minutes) and 11 destinations: small grocery store/supermarket, bakery, café, restaurant, physician, pharmacy, post office, bank/credit union, salon/barber shop, cemetery, recreation center. *Land use mix–destinations within 20-minute walk* was based on the number of shops accessible in ≤20 minutes. *Land use mix–proximity of destinations* was measured as the mean of all items. *Proximity of bus stop* was asked separately using the same question as it has been shown to be a significant determinant of active mobility [15].

To assess *walking infrastructure*, *cycling infrastructure*, *shared infrastructure (for walking and cycling)*, *street connectivity*, *aesthetics* and *traffic safety*, participants were asked ‘What does the environment/streetscape in your neighbourhood predominantly look like?’. They rated statements on a 4-point scale from ‘Strongly agree’ to ‘Strongly disagree’ (see S1 File for statements). For all these neighbourhood environmental attributes, subscale scores were calculated as the mean of all items per subscale.

### Statistical analysis

Respondents were excluded from this analysis if they were living in an institution (e.g. a care home) (n = 49), had missing data on all outcome variables (n = 32), or used a tricycle (n = 17). The final sample consisted of 2,144 older adults.

Absolute and relative frequencies of the study participants’ characteristics were calculated for the whole sample and stratified by sex. Descriptive statistics and tests of association (Chi-square, Wilcoxon or Kruskal-Wallis test) with each of the outcome variables were conducted for selected variables.

Binary logistic regression was used to identify associations of environmental attributes with the outcome variables ‘any cycling/e-biking’ and ‘frequent cycling/e-biking’ (≥ 3 days/week). Separate models for each of the aforementioned environmental attributes were calculated. Results are presented as Odds Ratios (OR) with corresponding 95% confidence intervals (CI). All environmental variables were treated as continuous variables and recoded so higher scores were expected to be related to more active mobility.

Linear regression models were used to examine associations of environmental attributes with the two outcomes ‘cycling amount’ and ‘e-biking amount’. A log-linear model was fitted to these variables as the empirical distributions in minutes/week were positively skewed. To account for overdispersion, a negative binomial regression with canonical log link function was performed. Results are presented as the exponential of L’Beta with corresponding 95% confidence intervals. It can be interpreted as the proportional increase or decrease in minutes per week cycling/e-biking associated with changes in the environmental attributes.

Multivariable logistic and linear regression analyzes were computed to analyze the association of environmental attributes with all six outcomes of active mobility behavior adjusted for age, sex, education (ISCED), income, partner status, self-rated health, area of residence and car ownership, as these variables were positively associated with one or more outcome variables in the previous tests of association (p-value <0.05).

Separate models were run to estimate interaction effects of environmental attributes with sex, age and mobility impairments. As this approach is exploratory, a probability level of 0.1 was set to not miss any weak but possible interaction effects. All environmental attributes with an interaction effect significant at a 0.1 probability level with any active mobility outcome in crude models are presented as stratified results. Adjusted models were run with the same covariates as in the main analysis, except for the moderator of mobility impairments. Here, self-rated health was excluded as covariate as it was correlated with mobility impairments.

All analyzes were carried out using SAS statistical software package version 9.4 (SAS Institute, Cary, NC, USA).

## Results

### Study population characteristics

Fifty-four percent of the whole sample was between 65–74 years old and 53% were men (see Table 1). Nearly the whole sample had a middle/high educational level (ISCED, 92%), lived in their own private home (97%), sometimes or always had access to a car (97%) and were of German origin (97%). The majority of the study participants (84%) owned some type of bicycle.

**Table 1:** Study characteristics for the total sample (N = 2144) and stratified by sex.

	Total			Men (N = 1088)			Women (N = 979)		
	N	n	%	N	n	%	N	n	%
**SOCIODEMOGRAPHICS**									
**Age**	2066			1086			979		
65–69		581	28.12		295	27.16		286	29.21
70–74		531	25.70		270	24.86		260	26.56
75–79		499	24.15		269	24.77		230	23.49
80+		455	22.02		252	23.20		203	20.74
**School education**	2132			1081			977		
Low		960	45.03		450	41.63		476	48.72
Middle		669	31.38		321	29.69		323	33.06
High		503	23.59		310	28.68		178	18.22
**ISCED**	2110			1071			965		
Low		163	7.73		32	2.99		127	13.16
Middle		1207	57.20		535	49.95		622	64.46
High		740	35.07		504	47.06		216	22.38
**Perceived financial situation**	2127			1084			969		
Very good/good		1352	63.56		697	64.30		612	63.16
Moderate/bad/very bad		775	36.44		387	35.70		357	36.84
**Income**	2074			1059			942		
Low		280	13.50		141	13.31		133	14.12
Middle		684	32.98		331	31.26		325	34.50
High		1110	53.52		587	55.43		484	51.38
**Living Situation**	2134			1085			975		
Own private home		2079	97.42		1065	98.16		942	96.62
Other		55	2.58		20	1.84		33	3.39
**Marital status**	2063			1086			976		
Married		1582	76.68		910	83.79		671	68.75
Single		138	6.69		78	7.18		60	6.15
Widowed		343	16.63		98	9.02		245	25.10
**Partner status**	2063			1086			976		
Partner		1613	78.19		934	86.00		678	69.47
No partner		450	21.81		152	14.00		298	30.53
**Country of birth**	2057			1083			973		
Germany		1926	93.63		1019	94.09		907	93.22
pre-war Germany		59	2.87		32	2.95		27	2.77
Europe		58	2.82		26	2.40		31	3.19
Outside Europe		14	0.68		6	0.55		8	0.82
**Area of residence**	2118			1075			967		
Medium-sized town		643	30.36		313	29.12		313	32.37
Larger small town		777	36.69		380	35.35		361	37.33
Small town		537	25.35		295	27.44		224	23.16
Rural ccommunity		161	7.60		87	8.09		69	7.14
**HEALTH**									
**Self-rated health**	2120			1075			969		
very good/good		1256	59.25		634	58.98		568	58.62
moderate/poor/very poor		864	40.75		441	41.02		401	41.38
**Walking aid**	2071			1059			937		
Yes		235	11.35		108	10.20		123	13.13
No		1836	88.65		951	89.80		814	86.87
**Mobility impairments**	2114			1079			960		
At least one		797	37.70		399	36.98		381	39.69
None		1317	62.30		680	63.02		579	60.31
**VEHICLE AVAILABILITY**	** **	** **	** **	** **	** **	** **	** **	** **	** **
**Car availability**	2097			1068			955		
Always		1872	89.27		1003	93.91		801	83.87
Sometimes		158	7.53		43	4.03		112	11.73
Never		67	3.20		22	2.06		42	4.40
**Bike availability**	2130			1082			971		
Conventional bicyle only		1031	48.40		537	49.63		450	46.34
E-bike only		384	18.03		196	18.11		169	17.40
Both types		380	17.84		218	20.15		151	15.55
None		335	15.73		131	12.11		201	20.70

The numbers of the total sample include participants with missing values in the variable sex.

More men than women had a high educational level according to ISCED and had more often access to a car or bicycle/e-bike. More women than men were widowed or had no partner.

### Frequency of cycling or e-biking for transport

Nearly two thirds of the study population engaged in any cycling for transport (65%, n = 1362) and one third in any e-biking (36%, n = 418) (see S2 Fig). The median time spent cycling and e-biking for transport was 120 minutes per week (n = 1231) and 150 minutes per week (n = 373), respectively. In this study, 42% (n = 881) of participants cycled three or more days a week for transport and 31% (n = 221) used an e-bike three or more days a week for this purpose.

### Socio-demographic characteristics and cycling

Engaging in any cycling/e-biking and cycling/e-biking frequency decreased with age and prevalence was higher in people with middle/high education (ISCED) (see Table 2). Any cycling/e-biking and frequent cycling/e-biking were associated with being male, perceiving a good/very good financial situation, being married, having a partner, having a good/very good health status, not using a walking aid, not having mobility impairments and always having a car available. Area of residence was only associated with any e-biking and frequent e-biking with higher amounts in larger small towns and small towns.

**Table 2:** Cycling or e-biking by sociodemographic characteristics, health status, and car availability.

	Any cycling for transport				Any e-biking for transport				Cycling frequency				E-biking frequency				Cycling amount				E-biking amount			
	* *	*Yes*		* *	* *	*Yes*		* *	* *	*≥3x/week*		* *	* *	*≥3x/week*		* *	*minutes/week*				*minutes/week*			
SOCIODEMOGRAPHICS	N	n	%	p-value^a^	N	n	%	p-value^a^	N	n	%	p-value^a^	N	n	%	p-value^a^	n	median	IQR	p-value^b^	n	median	IQR	p-value^b^
**Age**	2040				1135				2045	* *			693				1181				358			
65–69	576	425	73.8	< .0001	280	129	46.1	< .0001	574	267	46.5	< .0001	163	65	39.9	< .0001	394	135	240	0.3026	119	150	240	0.1473
70–74	525	375	71.4		262	112	42.8		526	251	47.7		150	58	38.7		343	120	180		101	120	210	
75–79	491	306	62.3		279	94	33.7		493	196	39.8		165	52	31.5		273	120	300		83	180	330	
80+	448	199	44.4		314	65	20.7		452	124	27.4		215	32	14.9		171	150	285		55	120	300	
**Sex**	2041				1136			* *	2046	* *			695				1181				358			
Male	1072	730	68.1	< .0001	566	224	39.6	0.0021	1079	470	43.6	0.0132	326	117	35.9	0.0013	659	135	240	0.3908	200	150	285	0.4536
Female	969	575	59.3		570	176	30.9		967	369	38.2		369	91	24.7		522	120	240		158	120	300	
**School education**	2102				1163			* *	2110	* *			714				1225				372			
Low	946	593	62.7	0.1766	578	225	38.9	0.0785	949	391	41.2	0.7383	388	129	33.3	0.3398	531	150	270	0.0084	199	160	300	0.1408
Middle	661	428	64.8		352	119	33.8	* *	664	272	41		209	60	28.7		383	135	240		106	150	220	
High	494	334	67.6		233	73	31.3		497	214	43.1		117	32	27.4		311	120	190		67	120	225	
**ISCED**	2080				1150				2089	* *			703				1220				372			
Low	158	72	45.6	< .0001	114	28	24.6	0.0207	163	42	25.8	< .0001	83	18	21.7	0.0798	68	135	250	0.0001	27	160	300	0.0419
Middle	1192	766	64.3		688	262	38.1	* *	1193	514	43.1		438	148	33.8		689	150	275		227	180	285	
High	730	507	69.5		348	125	35.9		733	315	43		182	54	29.7		463	120	180		118	120	210	
**Perceived financial situation**	2096				1161				2106	* *			713				1225				371			
Very good/good	1338	909	67.9	< .0001	711	282	39.7	0.0006	1335	596	44.6	0.0002	428	153	35.8	0.0008	827	120	240	0.178	254	150	300	0.2134
Moderate/bad/very bad	758	442	58.3		450	134	29.8		771	281	36.5		285	68	23.9		398	150	240		117	120	240	
**Income**	2044				1128				2054	* *			688				1201				366			
Low	276	160	58	0.0012	174	58	33.3	0.0792	275	104	37.8	0.1839	121	36	29.8	0.1944	144	150	300	0.0012	51	180	300	0.2031
Middle	670	416	62.1		378	124	32.8		676	276	40.8		232	65	28		377	160	280		104	180	300	
High	1098	749	68.2		576	227	39.4		1103	480	43.5		335	117	34.9		680	120	210		211	120	240	
**Marital status**	2037				1136				2042	* *			695				1178				358			
Married	1561	1050	67.3	< .0001	856	345	40.3	< .0001	1566	689	44	< .0001	510	183	35.9	< .0001	945	135	240	0.1517	306	150	300	0.8139
Single	136	78	57.4		68	10	14.7		136	38	27.9		40	5	12.5		72	120	240		10	135	270	
Widowed	340	173	50.9		212	45	21.2	* *	340	109	32.1		145	20	13.8		161	120	210		42	120	240	
**Partner status**	2037				1136				2042	* *			695				1178				358			
Partner	1591	1078	67.8	< .0001	860	347	40.4	< .0001	1596	699	43.8	< .0001	513	184	35.9	< .0001	969	135	240	0.1118	309	150	300	0.1276
No partner	446	223	50		276	53	19.2		446	137	30.7		182	24	13.2		209	120	240		49	120	240	
**Area of residence**	2088				1156			* *	2096	* *			707				1218				530			
Medium sized town	638	408	64	0.8651	331	101	30.5	0.0507	640	262	40.9	0.4277	209	44	21.1	0.0019	368	150	255	0.2819	136	155	300	0.5185
Larger small town	764	492	64.4		437	165	37.8		767	335	43.7		265	98	37		454	120	240		206	120	210	
Small town	527	346	65.7		301	120	39.9		531	208	39.2		187	63	33.7		309	120	240		151	150	300	
Rural community	159	99	62.3		87	27	31	* *	158	65	41.1		46	13	28.3		87	120	180		37	120	180	
**HEALTH**								* *		* *	* *	* *	* *	* *	* *	* *	* *	* *	* *	* *	* *	* *	* *	* *
**Self-rated health**	2088				1150				2098	* *			703				1222				368			
very good/good	1240	925	74.6	< .0001	583	268	46	< .0001	1239	625	50.4	< .0001	324	141	43.5	< .0001	839	150	240	0.0033	243	180	300	0.1193
moderate/poor/very poor	848	426	50.2		567	145	25.6		859	246	28.6		379	76	20.1		383	120	210		125	120	300	
**Walking aid**	2042				1116			* *	2049	* *			675				1211				365			
Yes	231	54	23.4	< .0001	211	34	16.1	< .0001	232	27	11.6	< .0001	167	17	10.2	< .0001	50	120	280	0.4021	30	150	280	0.4841
No	1811	1280	70.7		905	374	41.3	* *	1817	838	46.1		508	197	38.8		1161	120	240		335	150	300	
**Mobility impairments**	2082				1150				2093				706				1216				366			
At least one	783	369	47.1	< .0001	543	129	23.8	< .0001	790	219	27.7	< .0001	374	74	19.8	< .0001	326	120	290	0.367	109	180	330	0.0124
None	1299	973	74.9		607	281	46.3		1303	651	50		332	143	43.1		890	140	240		257	120	240	
**VEHICLE AVAILABILITY**								* *		* *	* *	* *		* *	* *	* *	* *	* *	* *	* *	* *	* *	* *	* *
**Car availability**	2067				1150				2077	* *			712				1208				373			
Always	1849	1243	67.2	< .0001	1002	396	39.5	< .0001	1856	790	42.6	0.0075	585	202	34.5	< .0001	1128	120	240	0.003	356	137.5	300	0.093
Sometimes	154	66	42.9		101	13	12.9		155	53	34.2		86	14	16.3		61	180	260		13	210	255	
Never	64	22	34.4		47	5	10.6	* *	66	18	27.3		41	3	7.32		19	300	510		4	390	258	

IQR = Interquartile range

^a^p-value of Chi^2^ test

^b^p-value of Wilcoxon or Kruskal-Wallis test.

Participants with good/very good self-rated health cycled more minutes per week, while participants with at least one mobility impairment had higher median minutes per week e-biking. Participants with no access to a car cycled/e-bicycled up to 180 more minutes per week for transport in the median than participants who sometimes or always had access to a car. It should be noted, however, that the group of people without access to a car was quite small. Overall, the median of minutes per week e-biking was always higher than in the group with any type of bicycle.

### Perceived environmental attributes and cycling

Most of the investigated environmental characteristics were associated with active mobility using any type of bicycle (see Table 3).

**Table 3:** Associations between perceived environmental attributes and any cycling/e-biking, frequency, and amount.

	Any cycling for transport		Any e-biking for transport				Cycling for transport ≥3x/week				E-biking for transport ≥3x/week				Cycling amount minutes/week				E-biking amount minutes/week			
Environmental attributes	n	OR (95% CI)	n	OR (95% CI)			n	OR (95% CI)			n	OR (95% CI)			n	exp(β) (95% CI)			n	exp(β) (95% CI)		
**Density**	1815		1006				1818				606				1074				329			
Model 0: Crude OR		1.00 (1.00–1.00)		1.00 (0.99–1.00)				1.00 (1.00–1.00)				0.99 (0.99–1.00)				1.00 (1.00–1.00)				1.00 (1.00–1.01)		
Model 1: adjusted OR		1.00 (1.00–1.00)		1.00 (1.00–1.01)				1.00 (1.00–1.01)				1.00 (0.99–1.01)				1.00 (1.00–1.00)				1.00 (1.00–1.00)		
**Walking infrastructure**	1763		974				1768				595				1044				319			
Model 0: Crude OR		1.11 (1.00–1.24) †		0.97 (0.84–1.11)				1.23 (1.10–1.37)				1.10 (0.90–1.35)				1.00 (0.94–1.07)				1.08 (0.96–1.22)		
Model 1: adjusted OR		1.14 (1.01–1.28)		1.05 (0.90–1.23)				1.24 (1.10–1.40)				1.25 (1.00–1.57) †				1.02 (0.95–1.09)				1.08 (0.95–1.22)		
**Cycling infrastructure**	1707		936				1709				562				1024				312			
Model 0: Crude OR		1.26 (1.10–1.43)		1.26 (1.06–1.50)				1.22 (1.07–1.38)				1.28 (1.02–1.61)				1.01 (0.93–1.09)				1.01 (0.88–1.17)		
Model 1: adjusted OR		1.22 (1.06–1.40)		1.26 (1.04–1.51)				1.17 (1.03–1.34)				1.26 (0.97–1.62)				1.01 (0.94–1.09)				1.00 (0.87–1.16)		
**Shared infrastructure**	1816		1008				1821				611				1072				330			
Model 0: Crude OR		1.17 (1.07–1.27)		1.17 (1.04–1.31)				1.18 (1.08–1.29)				1.25 (1.06–1.48)				1.00 (0.95–1.06)				0.96 (0.86–1.07)		
Model 1: adjusted OR		1.13 (1.03–1.24)		1.15 (1.01–1.30)				1.14 (1.04–1.25)				1.18 (0.98–1.42)				1.00 (0.95–1.06)				0.98 (0.88–1.09)		
**Street connectivity**	1768		978				1775				592				1047				320			
Model 0: Crude OR		1.28 (1.11–1.46)		1.22 (1.02–1.45)				1.42 (1.24–1.62)				1.34 (1.06–1.69)				1.14 (1.05–1.24)				1.23 (1.06–1.42)		
Model 1: adjusted OR		1.37 (1.18–1.59)		1.35 (1.11–1.63)				1.50 (1.30–1.73)				1.55 (1.18–2.04)				1.14 (1.05–1.24)				1.26 (1.09–1.47)		
**Aesthetics**	1668		916				1670				549				993				298			
Model 0: Crude OR		1.28 (1.12–1.47)		1.15 (0.96–1.37)				1.33 (1.17–1.52)				1.30 (1.01–1.66)				0.98 (0.91–1.06)				1.02 (0.88–1.18)		
Model 1: adjusted OR		1.25 (1.08–1.44)		1.18 (0.97–1.43)				1.29 (1.12–1.48)				1.34 (1.02–1.76)				1.00 (0.92–1.08)				1.03 (0.89–1.19)		
**Traffic safety**	1780		986				1785				593				1045				317			
Model 0: Crude OR		1.20 (1.04–1.39)		1.18 (0.97–1.44)				1.14 (0.99–1.32)				1.11 (0.86–1.44)				0.87 (0.80–0.95)				0.83 (0.70–0.97)		
Model 1: adjusted OR		1.08 (0.92–1.27)		1.08 (0.87–1.35)				1.05 (0.90–1.22)				0.9 3(0.69–1.26)				0.87 (0.80–0.95)				0.80 (0.68–0.96)		
**Land use mix—Proximity of destinations**	1858		1039				1862				630				1097				344			
Model 0: Crude OR		1.54 (1.37–1.71)		1.33 (1.16–1.54)				1.67 (1.50–1.87)				1.45 (1.19–1.76)				1.05 (0.99–1.12)				1.07 (0.95–1.19)		
Model 1: adjusted OR		1.48 (1.31–1.67)		1.34 (1.15–1.57)				1.62 (1.44–1.82)				1.39 (1.12–1.73)				1.07 (1.00–1.14) †				1.09 (0.97–1.23)		
**Land use mix—No. destinations within 20min walk**	1858		1039				1862				630				1097				344			
Model 0: Crude OR		1.10 (1.07–1.12)		1.07 (1.04–1.10)				1.11 (1.09–1.14)				1.08 (1.04–1.13)				1.01 (1.00–1.03) ‡				1.02 (0.99–1.04)		
Model 1: adjusted OR		1.09 (1.06–1.11)		1.07 (1.04–1.10)				1.11 (1.08–1.13)				1.07 (1.03–1.12)				1.02 (1.00–1.03) ‡				1.02 (1.00–1.05) †		
**Proximity of bus stop**	1858		1039				1862				630				1097				344			
Model 0: Crude OR		1.20 (1.10–1.32)		1.12 (0.99–1.26)				1.17 (1.07–1.29)				1.11 (0.94–1.32)				0.97 (0.91–1.03)				0.95 (0.86–1.06)		
Model 1: adjusted OR		1.15 (1.03–1.27)		1.09 (0.96–1.25)				1.13 (1.02–1.25)				1.09 (0.90–1.32)				0.97 (0.92–1.03)				0.96 (0.86–1.07)		

OR = Odds Ratio; exp(ß) = exponentiated L’Beta; CI = Confidence Interval.

Model 0: Separate models with single environmental attributes; Model 1: Additionally adjusted for age, sex, education (ISCED), equivalized disposable income, partner status, self-rated health, area of residence and car-ownership.

‡p<0.05

†p≥0.05.

*Street connectivity* was positively associated with all of the six cycling outcomes in adjusted models. Each unit increase in perceived street connectivity was associated with a 35–55% higher chance of active mobility (any or frequent cycling/e-biking) and 14–26% more minutes per week cycling/e-biking for transport.

*Proximity of destinations* showed the strongest positive association with any cycling and frequent cycling compared to the other environmental attributes: each unit increase in proximity raised the chance to engage in any cycling by 48% and in cycling ≥3 times per week by 62%. Moreover, each additional destination within 20 minutes walking distance increased the likelihood of cycling/e-biking and frequent cycling/e-biking by 7–11% and cycling minutes per week by 2%. Proximity of a bus stop was positively associated with any cycling and frequency of cycling for transport but not with any e-biking or frequent e-biking.

*Walking infrastructure* was positively associated with any cycling and cycling frequency. Cycling infrastructure and shared infrastructure were positively associated with any cycling/e-biking and frequent cycling but not with frequent e-biking after adjustment. There was no association for minutes cycling or e-biking with land use mix, infrastructure and proximity of a bus stop.

*Aesthetics* showed positive associations with engaging in any cycling and frequent cycling/e-biking but not with cycling/e-biking amount.

*Traffic safety* was not associated with any cycling/e-biking and cycling/e-biking frequency in adjusted models, and was in fact inversely associated with cycling and e-biking amount; each unit increase in the perception of traffic safety meant a 13% reduction in cycling and 20% reduction in e-biking minutes per week in the adjusted models.

No association between residential density and any of the six cycling outcomes could be observed.

### Interaction effects

In stratified analysis some of the observed associations between the built environment attributes and cycling outcomes were stronger or only apparent in some groups. Among men, cycling amount was positively associated with proximity of destinations and number of close destinations, but not among women (see Table 4 in S1 Table). Among women, there were inverse associations for proximity of a bus stop with cycling amount and for traffic safety with cycling and e-biking amount.

For the younger age group (70–74 years), cycling infrastructure was positively associated with e-biking amount. The older age group (75–79 years) showed a positive association between aesthetics and e-biking amount. In contrast to the younger age groups and the main analysis (Table 3), traffic safety among 75-79-year-olds in the sample was positively associated with cycling frequency, with a 52% higher chance of frequent cycling with each unit increase in traffic safety. The association among the oldest age group pointed in the same direction but was not significant.

An inverse association for traffic safety with cycling amount could be observed among people with at least one mobility impairment. This group was also 2.4 times more likely to cycle with their e-bike frequently with each unit increase in street connectivity. In contrast, the other environmental attributes were only associated with cycling among people without mobility impairments: each unit increase in aesthetics was positively associated with any cycling, shared infrastructure with any cycling/e-biking, proximity of a bus stop with any e-biking, and walking infrastructure with e-biking amount.

## Discussion

Our study is one of the first to investigate the association between environmental attributes and cycling/e-biking for transport in older adults. Overall, the estimates point to the expected direction of a positive relationship between a cycling-friendly built environment and cycling for transport. The most important environmental attributes were street connectivity and land use mix. Only street connectivity led to an increase of the amount of cycling and e-biking. For e-biking, fewer environmental attributes showed an association compared to cycling of any kind. Unexpectedly, traffic safety was not associated with four out of the six cycling outcomes and was even inversely associated with minutes spent cycling or e-biking for transport. Density was not associated with any of the outcome variables investigated. Explorative analysis indicated that especially mobility impairments, but also sex and age might play a role as effect modifier.

The prevalence of frequent cycling (≥3 times/week) was three times higher in our study sample than in the general population of this age cohort in Germany [9]. However, the pattern that men cycle slightly more frequently than women and highly educated groups more than those with middle or low education is the same in the general population [9]. A German transport survey has shown that most e-bike owners in Germany are older people living in medium-sized and small-town or rural areas, which might explain the high percentage of e-bike owners in our study. The median number of minutes e-biking was higher in our study sample compared to cycling of any type, which is in line with previous investigations [9,40]. Most interestingly, e-biking amount was significantly higher among those with at least one mobility impairment. This confirms results from other studies which suggest that e-biking enables people with decreasing physical health to continue cycling [22].

In our main analysis, better neighborhood street connectivity showed the strongest association with cycling. Street connectivity indicates short distances between intersections and therefore probably reachable destinations, which coincides with the second most important factor in this study, ’proximity of destinations’. Other studies have concluded that street connectivity is a facilitator rather than an essential determinant of cycling. They confirm our finding that proximity to destinations visited daily is a consistent correlate of both older adults’ total active mobility behavior [15] and cycling [27]. The same has been found for adults’ total active mobility [41] and cycling [42]. However, we found no association between proximity of destinations and an increased number of minutes cycled per week. This is in line with findings among older adults from Belgium [43]. One can assume that the closer the destinations are, the fewer minutes people would cycle. The fact that the estimators in our study nevertheless point in a positive direction indicates that people who perceive accessible destinations in their residential environment tend to cycle for more minutes because they visit them more frequently. This supports our result concerning the number of destinations within a short distance, specifically that more minutes per week are spent cycling when there are more shops nearby. In the case of proximity to a bus stop, e-biking alone was not associated, although cycling generally showed a positive association. Previous research suggests that people with e-bikes might be worried to lock their e-bikes at the bus stop because of fear of theft [22,44].

The positive relationship between cycling infrastructure (defined as a cycling path, walking path or shared paths) and any cycling and frequent cycling is in line with evidence on cycling behavior in adults [27,45,46]. In a recent study of older adults in Belgium, the authors found that the type of cycle path (i.e. separated vs. no cycling infrastructure) was the most important attribute among all participants and among those with the highest levels of mobility impairments and lowest levels of cycling [17]. Improving the availability and quality of walking and cycling facilities may therefore benefit those older adults that are most in need of cycling infrastructure. However, we found a positive relationship between walking or shared infrastructure and cycling for transport. It is important, however, that infrastructure to encourage cycling does not inadvertently discourage walking. Cycling on the footpath, for example, is not practical as this can produce conflict and safety issues with walkers who also need safe and convenient space for active mobility [18]. In comparison, frequent e-biking showed a weak association with cycling and shared infrastructure, and no association with walking infrastructure. In fact, e-bike users in a qualitative study reported they feel more safe cycling on the street with other vehicles that are driving at a similar speed [22].

The negative association of active modes of transport with traffic safety has also been reported for total minutes of walking in adults in a study comparing 17 countries [42], for daily cycling for transport in Belgian older females [47], and for total active mobility in an older population from South Africa [48]. The lack of an association between traffic safety and the other cycling outcomes investigated in our study is consistent with a study examining Brazilian older adults’ total active mobility behavior [49]. This result might be explained by the cross-sectional nature of this study in terms of reported perception of environmental attributes, which suggests that people who cycle frequently are more aware of traffic safety problems. Qualitative studies with older adults point to the importance of traffic safety when walking [13,18]. Although we found no association in the overall sample, we can confirm a relationship for the older age groups (75+) in our study who cycled/e-bicycled more frequently with increased traffic safety, which further highlights the importance of managing safety concerns for age-friendly environments.

Previous results on the relationship between aesthetics and active mobility have been mixed, which may be explained by different measurement approaches [27,50,51]. In contrast to another study [27], we found no interaction between neighborhood aesthetics and sex. Concerning e-biking, our results showed that aesthetics were associated with frequent e-biking but not with any e-biking, which suggests that an aesthetically pleasing environment might support more frequent e-bicycle use but does not influence uptake. Furthermore, our results show an interaction effect with younger age (<75 years) on more minutes of e-biking. One may conclude, that at some point–given experience and routine with age–cycling in aesthetically pleasing environments might become less important for time spent cycling per week in older age while traffic safety issues come into focus.

No association between density and any of the active mobility outcomes could be observed in our study. Results of studies using the same instrument are mixed [27,42]. We believe this to be an artefact of the density measure we used. This instrument was developed and validated in urban areas with more diverse housing [38]. Our study area is characterized by many single-family residences and row houses of 1–3 stories. Therefore, residential density in our study might be quite homogenous. In future studies, a valid assessment of residential density applicable for more rural areas should be considered.

### Limitations

This study is not without limitations. The cross-sectional nature of this study does not allow conclusions to be drawn about the causality of the associations. Furthermore, older adults tend to overreport their physical activity using the IPAQ questionnaire [52], which has to be considered. However, we assume that this potential misclassification is independent of the built environment. Also, questions on the perceived environment used in this study might be less relevant in extremely rural areas, where no sidewalks at all are present and street trees, lighting, benches and litter bins are less common. This might have led to missing values or misclassification and limiting the extent to which unfavorable environments could be characterized. Lastly, compared to the general population of the age group 65 years and older in the state of Lower Saxony, there were more men in our sample (53% vs. 43%) more people were married (77% vs. 60%) [53,54] and the proportion of very good/good self-rated health was higher (59% vs. 47%) [55].

### Implications

As proximity of destinations seems to be one of the most important correlates of cycling, it is important to preserve facilities and services. This is especially important in smaller communities, where even investments in better cycling infrastructure are unlikely to offset the negative effect that longer distances can have on cycling. Proximity of destinations was more important for cycling in older adults than a variety of shops and services in our study. Future studies should investigate which kind of shops and services are most important to ensure they are in a reachable distance in order to reduce car dependency and enable elderly people to live autonomously. This could help communities with a small population and low purchasing power to prioritize on preserving or locate certain services.

Providing cycling facilities seems desirable to increase bicycle use among older adults. Although previous studies have mostly been conducted in larger cities [46,56], this study has confirmed the positive effect of cycling and shared infrastructure as well as walking infrastructure on cycling and cycling frequency for smaller towns. However, knowledge about the status-quo of infrastructure in more differentiated town sizes and its impacts on cycling levels is still limited. Even more important than cycling infrastructure was street connectivity. Although the overall street network is hard to change, shortcuts for cyclists might be included in transport and settlement planning to increase street connectivity. Furthermore, the study results suggest that aesthetically pleasing environments, for example those with trees, gardens and attractive buildings, may favor cycling.

Traffic safety has been reported in qualitative studies to be a crucial determinant of active mobility [8,18], and measures improving safety have been found to be effective in encouraging cycling in intervention studies [40]. In this study, however, this association was only found within older age groups. We suspect this is a result of the entanglement of perception of traffic safety with rising cycling frequency and experience in our data, and recommend that future studies investigate how to assess perceived traffic safety quantitatively and in greater detail to better understand this issue.

## Conclusions

This study emphasizes opportunities to promote cycling through shaping aspects of the environment, especially through maximizing the proximity to destinations, street connectivity, and cycling infrastructure. This is actually in line with the recently adopted national strategy for the promotion of cycling in Germany. The National Cycling Plan 3.0 aims to double the kilometers travelled by bike or e-bike by the year 2030 and to make cycling both safer and more attractive. A key objective is to create a seamless network of safe, intuitive and convenient cycle paths and streets that enable all age groups to cycle more frequently. The importance of accessible destinations is less in the foreground. But the need for an integrated approach to city and transport planning and a promotion of a “city of short distances” is acknowledged [57].

Further research is needed to identify beneficial factors for e-biking. In our study, people over the age of 65 and with mobility impairments used an e-bike, which may support them to stay active and mobile in their neighborhood and even cover longer distances. However, awareness of e-bikes among all road users has to be promoted if e-bike users are supposed to cycle on the street. Street space in smaller communities or historic centers is limited and it is not always possible to set up a separated cycle path that is wide enough to allow for cyclists travelling at different speeds. For public health, the current findings underline the importance of investigating not only the amount of physical activity, but also any engagement in active mobility regardless of the quantity. Any cycling at all can make all the difference for independent living in old age. The coronavirus pandemic since 2020 is a direct reminder that health is a public good worth protecting. Not only individual pre-existing conditions, but also the living conditions of older people in the community influence how older people can cope with health burdens. Healthy living environments are an important resource that can be shaped by the municipalities.

## Supporting information

S1 Fig Flow chart of survey response rates. (DOCX)

S2 Fig Creation and definition of outcomes and respective reference categories. (DOCX)

S1 Table Results of interaction effects. (DOCX)

S1 File Statements on the assessed environmental attributes (based on the NEWS questionnaire). (DOCX)
