# Supplementary figures and images for: The relationship between perceived built environment and cycling or e-biking for transport among older adults–a cross-sectional study
Source: PLoS One. 2022 May 3;17(5):e0267314. doi: 10.1371/journal.pone.0267314 (PMC9064114; doi:10.1371/journal.pone.0267314)

**S1 Fig: Flow chart of survey response rates**


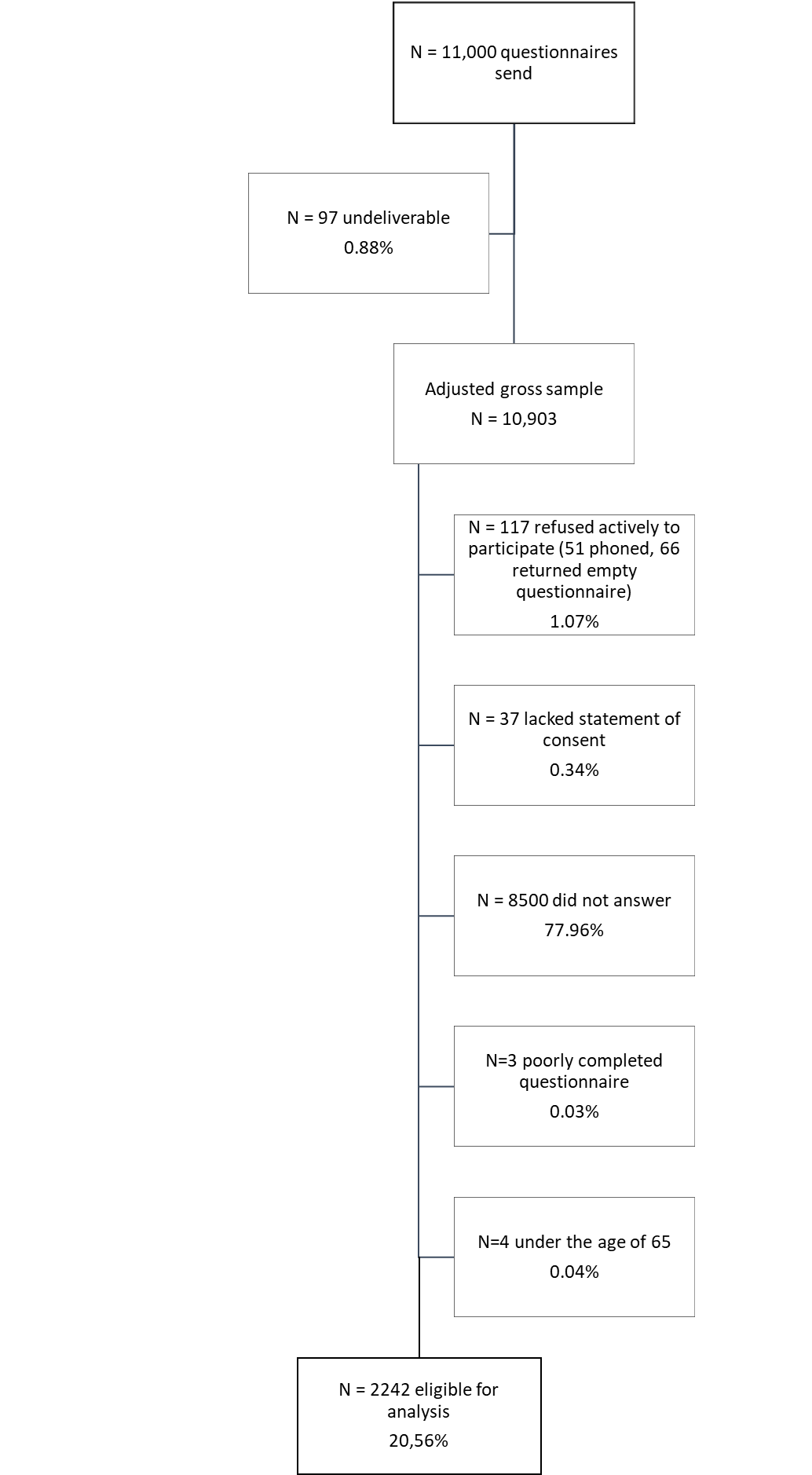

Supplement: S1 Fig — (DOCX) [file pone.0267314.s001.docx]
