# Supplementary material for: The relationship between perceived built environment and cycling or e-biking for transport among older adults–a cross-sectional study
Source: PLoS One. 2022 May 3;17(5):e0267314. doi: 10.1371/journal.pone.0267314 (PMC9064114; doi:10.1371/journal.pone.0267314)
Supplement: S2 Fig — (DOCX) [file pone.0267314.s002.docx]

**S2 Fig:** **Creation and definition of outcomes and respective reference categories**


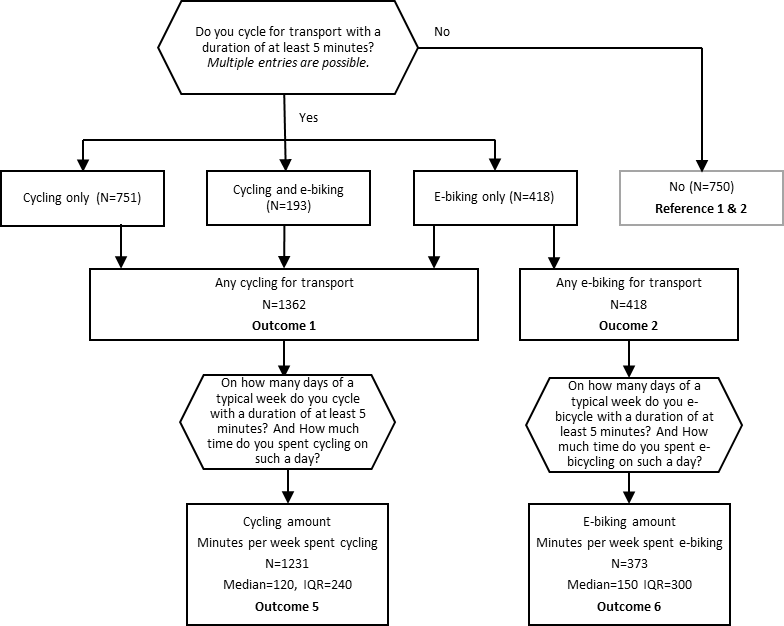


Fig S2.1: Creation and definition of outcomes 1 and 2 (any cycling and any e- biking) and 5 and 6 (cycling amount minutes/week and e-biking amount minutes/week) and respective reference categories.


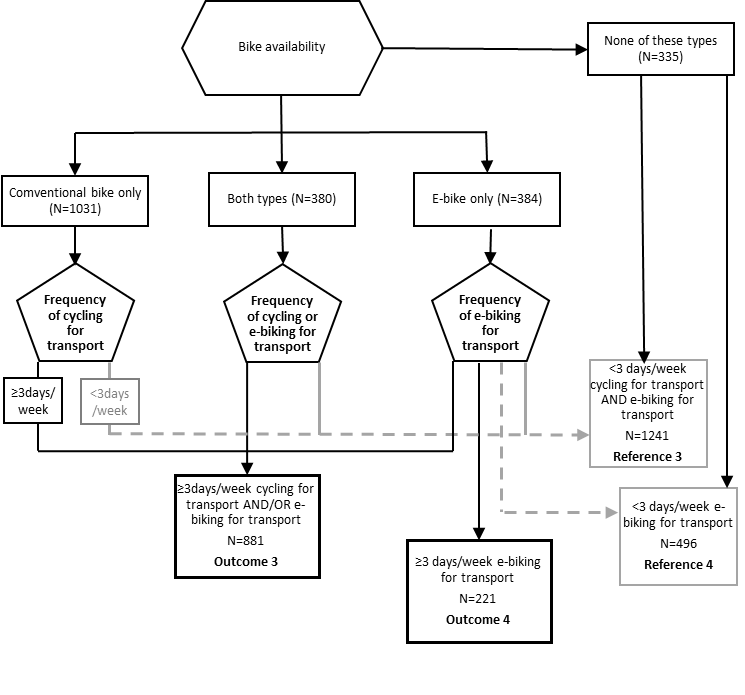


Fig S2.2: Creation and definition of outcomes 3 and 4 (cycling ≥ 3 days/week and e-biking ≥ 3 days/week) and respective reference categories
