# Supplementary material for: The relationship between perceived built environment and cycling or e-biking for transport among older adults–a cross-sectional study
Source: PLoS One. 2022 May 3;17(5):e0267314. doi: 10.1371/journal.pone.0267314 (PMC9064114; doi:10.1371/journal.pone.0267314)
Supplement: S1 Table — (DOCX) [file pone.0267314.s003.docx]

**S4 Table: Results of interaction effects**

| **Table 4: Effect modification by sex, age, and mobility impairments of the association of perceived environmental attributes with any cycling/e-biking for transport, frequency, and amount.** | | | | | | | | | | |
| --- | --- | --- | --- | --- | --- | --- | --- | --- | --- | --- |
|  |  | Model 0: Crude | | | |  | Model 1: Adjusted | | |  |
| *Any cycling for transport* |  | n | OR | 95% CI | |  | OR | 95% CI | |  |
| **Density*sex** |  |  |  |  |  |  |  |  |  |  |
| Male |  | 956 | 1.00 | 1.00 | 1.01 |  | 1.00 | 1.00 | 1.01 |  |
| Female |  | 859 | 1.00 | 0.99 | 1.00 |  | 1.00 | 0.99 | 1.00 |  |
| **Density*impairments** |  |  |  |  |  |  |  |  |  |  |
| At least 1 mobility impairment |  | 666 | 1.00 | 0.99 | 1.00 |  | 1.00 | 0.99 | 1.00 |  |
| None |  | 1134 | 1.00 | 1.00 | 1.01 |  | 1.01 | 1.00 | 1.01 |  |
| **Shared infrastructure*impairments** |  |  |  |  |  |  |  |  |  |  |
| At least 1 mobility impairment |  | 670 | 0.98 | 0.85 | 1.12 |  | 0.94 | 0.81 | 1.09 |  |
| None |  | 1131 | 1.28 | 1.13 | 1.45 |  | 1.27 | 1.12 | 1.43 |  |
| **Aesthetics*impairments** |  |  |  |  |  |  |  |  |  |  |
| At least 1 mobility impairment |  | 614 | 1.06 | 0.87 | 1.31 |  | 1.11 | 0.88 | 1.40 |  |
| None |  | 1041 | 1.36 | 1.13 | 1.64 |  | 1.38 | 1.14 | 1.67 |  |
| *Any e-bicycling for transport* |  |  |  |  |  |  |  |  |  |  |
| **Density*sex** |  |  |  |  |  |  |  |  |  |  |
| Male |  | 498 | 1.00 | 1.00 | 1.01 |  | 1.01 | 1.00 | 1.02 |  |
| Female |  | 508 | 0.99 | 0.98 | 1.00 |  | 0.99 | 0.99 | 1.00 |  |
| **Walking infrastructure*age** |  |  |  |  |  |  |  |  |  |  |
| 65-69 |  | 258 | 1.18 | 0.90 | 1.56 |  | 1.20 | 0.89 | 1.60 |  |
| 70-74 |  | 227 | 0.87 | 0.65 | 1.16 |  | 0.89 | 0.64 | 1.24 |  |
| 75-79 |  | 241 | 1.19 | 0.88 | 1.61 |  | 1.38 | 0.98 | 1.93 |  |
| 80+ |  | 248 | 0.71 | 0.52 | 0.97 |  | 0.80 | 0.56 | 1.15 |  |
| **Shared infrastructure*impairments** |  |  |  |  |  |  |  |  |  |  |
| At least 1 mobility impairment |  | 464 | 0.97 | 0.80 | 1.18 |  | 0.91 | 0.73 | 1.12 |  |
| None |  | 535 | 1.30 | 1.11 | 1.53 |  | 1.31 | 1.11 | 1.55 |  |
| **Proximity of a bus stop*impairments** |  |  |  |  |  |  |  |  |  |  |
| At least 1 mobility impairment |  | 478 | 0.91 | 0.75 | 1.10 |  | 0.84 | 0.68 | 1.05 |  |
| None |  | 551 | 1.17 | 0.99 | 1.38 |  | 1.25 | 1.04 | 1.49 |  |
| *Cycling for transport ≥3x/week* |  |  |  |  |  |  |  |  |  |  |
| **Traffic safety*age** |  |  |  |  |  |  |  |  |  |  |
| 65-69 |  | 522 | 0.97 | 0.74 | 1.25 |  | 0.93 | 0.71 | 1.23 |  |
| 70-74 |  | 467 | 0.89 | 0.67 | 1.19 |  | 0.80 | 0.59 | 1.08 |  |
| 75-79 |  | 423 | 1.50 | 1.11 | 2.04 |  | 1.52 | 1.11 | 2.10 |  |
| 80+ |  | 373 | 1.31 | 0.92 | 1.87 |  | 1.28 | 0.87 | 1.89 |  |
| **Density*impairments** |  |  |  |  |  |  |  |  |  |  |
| At least 1 mobility impairment |  | 669 | 1.00 | 0.99 | 1.00 |  | 1.00 | 0.99 | 1.00 |  |
| None |  | 1135 | 1.00 | 1.00 | 1.01 |  | 1.00 | 1.00 | 1.01 |  |
| *E-bicycling for transport ≥3x/week* |  |  |  |  |  |  |  |  |  |  |
| **Traffic safety*age** |  |  |  |  |  |  |  |  |  |  |
| 65-69 |  | 149 | 0.72 | 0.44 | 1.19 |  | 0.57 | 0.32 | 1.01 |  |
| 70-74 |  | 129 | 1.04 | 0.58 | 1.86 |  | 0.82 | 0.41 | 1.64 |  |
| 75-79 |  | 146 | 1.43 | 0.85 | 2.40 |  | 1.59 | 0.88 | 2.87 |  |
| 80+ |  | 169 | 1.18 | 0.65 | 2.15 |  | 1.06 | 0.50 | 2.25 |  |
| **Street connectivity*impairments** |  |  |  |  |  |  |  |  |  |  |
| At least 1 mobility impairment |  | 303 | 1.77 | 1.20 | 2.61 |  | 2.36 | 1.47 | 3.79 |  |
| None |  | 284 | 1.05 | 0.76 | 1.45 |  | 1.17 | 0.82 | 1.66 |  |
|  |  |  |  |  |  |  |  |  |  |  |

**S4 Table continued**

|  |  | Model 0: Crude | | | |  | Model 1: Adjusted | | |  |
| --- | --- | --- | --- | --- | --- | --- | --- | --- | --- | --- |
| *Cycling amount min/week* |  | n | exp(β) | 95% CI | |  | exp(β) | 95% CI | |  |
| **Traffic safety*sex** |  |  |  |  |  |  |  |  |  |  |
| Male |  | 593 | 0.94 | 0.84 | 1.06 |  | 0.96 | 0.85 | 1.08 |  |
| Female |  | 452 | 0.80 | 0.70 | 0.90 |  | 0.80 | 0.71 | 0.91 |  |
| **Land use mix proximity*sex** |  |  |  |  |  |  |  |  |  |  |
| Male |  | 616 | 1.14 | 1.04 | 1.25 |  | 1.15 | 1.05 | 1.26 |  |
| Female |  | 482 | 0.96 | 0.87 | 1.06 |  | 0.96 | 0.88 | 1.06 |  |
| **Land use mix destinations *sex** |  |  |  |  |  |  |  |  |  |  |
| Male |  | 616 | 1.03 | 1.01 | 1.05 |  | 1.03 | 1.01 | 1.05 |  |
| Female |  | 482 | 1.00 | 0.98 | 1.02 |  | 1.00 | 0.98 | 1.02 |  |
| **Proximity of a bus stop*sex** |  |  |  |  |  |  |  |  |  |  |
| Male |  | 616 | 1.06 | 0.98 | 1.15 |  | 1.07 | 0.99 | 1.16 |  |
| Female |  | 482 | 0.87 | 0.80 | 0.95 |  | 0.87 | 0.79 | 0.95 |  |
| **Density*impairments** |  |  |  |  |  |  |  |  |  |  |
| At least 1 mobility impairment |  | 285 | 1.00 | 0.99 | 1.00 |  | 1.00 | 1.00 | 1.00 |  |
| None |  | 781 | 1.00 | 1.00 | 1.00 |  | 1.00 | 1.00 | 1.00 |  |
| **Traffic safety*impairments** |  |  |  |  |  |  |  |  |  |  |
| At least 1 mobility impairment |  | 279 | 0.76 | 0.65 | 0.90 |  | 0.78 | 0.66 | 0.93 |  |
| None |  | 757 | 0.93 | 0.84 | 1.03 |  | 0.94 | 0.85 | 1.05 |  |
| **Proximity of a bus stop*impairments** |  |  |  |  |  |  |  |  |  |  |
| At least 1 mobility impairment |  | 293 | 0.88 | 0.79 | 0.99 |  | 0.93 | 0.83 | 1.04 |  |
| None |  | 795 | 1.01 | 0.94 | 1.08 |  | 1.01 | 0.94 | 1.08 |  |
| *E-bicycling amount min/week* |  |  |  |  |  |  |  |  |  |  |
| **Density*sex** |  |  |  |  |  |  |  |  |  |  |
| Male |  | 183 | 1.00 | 0.99 | 1.00 |  | 1.00 | 0.99 | 1.00 |  |
| Female |  | 146 | 1.01 | 1.00 | 1.01 |  | 1.01 | 1.00 | 1.02 |  |
| **Traffic safety*sex** |  |  |  |  |  |  |  |  |  |  |
| Male |  | 181 | 0.96 | 0.77 | 1.21 |  | 1.00 | 0.80 | 1.26 |  |
| Female |  | 136 | 0.70 | 0.55 | 0.89 |  | 0.66 | 0.51 | 0.85 |  |
| **Cycling infrastructure*age** |  |  |  |  |  |  |  |  |  |  |
| 65-69 |  | 105 | 1.10 | 0.81 | 1.49 |  | 1.18 | 0.87 | 1.61 |  |
| 70-74 |  | 92 | 1.25 | 0.98 | 1.61 |  | 1.33 | 1.02 | 1.75 |  |
| 75-79 |  | 75 | 0.84 | 0.64 | 1.10 |  | 0.85 | 0.64 | 1.13 |  |
| 80+ |  | 40 | 0.86 | 0.63 | 1.17 |  | 0.89 | 0.64 | 1.23 |  |
| **Aesthetics*age** |  |  |  |  |  |  |  |  |  |  |
| 65-69 |  | 101 | 1.10 | 0.81 | 1.49 |  | 1.20 | 0.89 | 1.61 |  |
| 70-74 |  | 90 | 1.31 | 1.00 | 1.72 |  | 1.39 | 1.04 | 1.84 |  |
| 75-79 |  | 71 | 0.89 | 0.68 | 1.17 |  | 0.92 | 0.70 | 1.20 |  |
| 80+ |  | 36 | 0.79 | 0.56 | 1.12 |  | 0.73 | 0.53 | 1.01 |  |
| **Traffic safety*age** |  |  |  |  |  |  |  |  |  |  |
| 65-69 |  | 111 | 0.70 | 0.53 | 0.94 |  | 0.73 | 0.53 | 1.01 |  |
| 70-74 |  | 90 | 0.78 | 0.58 | 1.05 |  | 0.73 | 0.52 | 1.02 |  |
| 75-79 |  | 71 | 1.08 | 0.77 | 1.51 |  | 1.04 | 0.71 | 1.52 |  |
| 80+ |  | 45 | 0.88 | 0.57 | 1.33 |  | 1.09 | 0.70 | 1.70 |  |
| **Walking infrastructure*impairments** |  |  |  |  |  |  |  |  |  |  |
| At least 1 mobility impairment |  | 293 | 0.88 | 0.79 | 0.99 |  | 0.96 | 0.77 | 1.19 |  |
| None |  | 795 | 1.01 | 0.94 | 1.08 |  | 1.19 | 1.03 | 1.37 |  |
|  |  |  |  |  |  |  |  |  |  |  |
| OR=Odds Ratio; exp(ß)=exponentiated L'Beta; CI=Confidence Interval | | | | | |  |  |  |  |  |
| Model 0: Separate models with single environmental attributes; Model 1: additionally adjusted for age (if not a moderator), sex (if not a moderator), education (ISCED), equivalized disposable income, partner status, self-rated health (if not mobility impairments as moderator), area of residence and car-ownership | | | | | | | | | | |
