# Supplementary material for: The relationship between perceived built environment and cycling or e-biking for transport among older adults–a cross-sectional study
Source: PLoS One. 2022 May 3;17(5):e0267314. doi: 10.1371/journal.pone.0267314 (PMC9064114; doi:10.1371/journal.pone.0267314)
Supplement: S1 File — (DOCX) [file pone.0267314.s004.docx]

**S3 File: Statements on the assessed environmental attributes (based on the NEWS questionnaire)**

*Walking infrastructure*

‘There are sidewalks on most of the streets in my neighborhood’

‘The sidewalks are well maintained (paved, even, and few potholes)’

‘The sidewalks are wide enough’

*Cycling infrastructure*

‘There are cycle paths on most of the streets in my neighborhood’

‘The cycle paths are well maintained (paved, even, and few potholes)’

‘The cycle paths are wide enough’

*Shared infrastructure*

‘There are shared paths for walking and cycling’

*Street connectivity*

‘There are many four-way intersections’

‘The distance between intersections is usually short (100 meters or less; the length of a soccer field or less)’

‘There are many alternative routes for getting from place to place (I don't have to go the same way every time)’

*Aesthetics*

‘There are trees along the streets in my neighborhood’

‘Trees give shade for the sidewalks in my neighborhood’

‘There are many interesting things to look at while walking in my neighborhood’

‘My neighborhood is generally free from litter’

‘There are many attractive natural sights in my neighborhood (such as (front) gardens, landscaping, views)’

There are attractive buildings/homes in my neighborhood

*Traffic safety*

‘There is a lot of traffic along the street I live in’

‘The speed of traffic on the street where I live is usually fast’

‘Most drivers exceed the posted speed limits while driving in my street’

‘I like to walk along the street where I live’

‘On this street, I feel safe from crime’

‘On this street, I feel safe from road accidents’

‘Crossing the road is safe for pedestrians’
